# Role of Regulatory T Cells in Regulating Fetal-Maternal Immune Tolerance in Healthy Pregnancies and Reproductive Diseases

**DOI:** 10.3389/fimmu.2020.01023

**Published:** 2020-06-26

**Authors:** Ning Huang, Hongbin Chi, Jie Qiao

**Affiliations:** Center for Reproductive Medicine, Department of Obstetrics and Gynecology, Peking University Third Hospital, Beijing, China

**Keywords:** regulatory T cells, pregnancy, steroidogenesis, endometriosis, primary unexplained infertility, recurrent spontaneous abortion, preeclampsia

## Abstract

Regulatory T cells (Tregs) are a specialized subset of T lymphocytes that function as suppressive immune cells and inhibit various elements of immune response *in vitro* and *in vivo*. While there are constraints on the number or function of Tregs which can be exploited to evoke an effective anti-tumor response, sufficient expansion of Tregs is essential for successful organ transplantation and for promoting tolerance of self and foreign antigens. The immune-suppressive property of Tregs equips this T lymphocyte subpopulation with a pivotal role in the establishment and maintenance of maternal tolerance to fetal alloantigens, which is necessary for successful pregnancy. Elevation in the level of pregnancy-related hormones including estrogen, progesterone and human chorionic gonadotropin promotes the recruitment and expansion of Tregs, directly implicating these cells in the regulation of fetal-maternal immune tolerance. Current studies have provided evidence that a defect in the number or function of Tregs contributes to the etiology of several reproductive diseases, such as recurrent spontaneous abortion, endometriosis, and pre-eclampsia. In this review, we provide insight into the underlying mechanism through which Tregs contribute to pregnancy-related immune tolerance and demonstrate the association between deficiencies in Tregs and the development of reproductive diseases.

## Introduction

Regulatory T cells (Tregs), a key subset of T lymphocytes, play a critical role in regulating the immune response and maintaining immune tolerance both in physiological and pathological processes. Many studies have shown that Tregs are compromised in patients with autoimmune diseases as well as in patients with graft-versus-host disease after receiving transplanted organs (1), however, these cells are activated to promote tumor growth and progression, leading to the failure of immunotherapies in cancer (2). Defects in the number of Tregs and their suppressive activity are involved in the development of various systemic or organ-specific autoimmune diseases, including thyroiditis (3), gastritis (4), type I diabetes (T1D) (5), systemic lupus erythematosus (SLE) (6), multiple sclerosis (MS) (7), rheumatoid arthritis (RA) (8), and inflammatory bowel disease (IBD) (9).

During the course of pregnancy, the mother's systemic immune system is altered to tolerate the fetus, who expresses paternal major histocompatibility complex antigens. Many studies have supplied multiple lines of evidence that Tregs possess specific characteristics for preventing the development of a maternal immune response against the fetus and maintaining fetal-maternal tolerance. First, the proportion of Tregs in peripheral blood is significantly increased during pregnancy in both women and mice, and there is a specific recruitment of Tregs from maternal peripheral blood to the fetal-maternal interface, leading to a higher proportion of Tregs in the placental decidua than in the peripheral blood (10). Furthermore, a decreased proportion of Tregs has been proposed to be associated with pregnancy-related complication such as recurrent spontaneous abortion and pre-eclampsia (11–13). Second, antibody-mediated depletion of CD25^+^ Tregs has been shown to cause implantation failure in allogeneic mated mice (14). Conversely, the adoptive transfer of Tregs attenuates the high abortion rates in the well-studied CBA/J × DBA/2J abortion-prone murine model (15).

Pregnancy is a physiological process greatly dependent on immune tolerance, which is regulated by the number of Tregs and their suppressive activity. This review of the current literature describes the role played by Tregs in regulating fetal-maternal immune tolerance. Furthermore, we demonstrate the relationship between a deficiency of Tregs and pregnancy-related complications, with the aim of identifying the mechanisms through which Tregs maintain fetal-maternal immune homeostasis, thus providing a potential target for treating pregnancy-related complications.

### Differentiation and Immunosuppressive Function of Tregs

Tregs are divided into two populations, namely natural regulatory T cells (nTregs) and inducible regulatory T cells (iTregs). NTregs originate from the thymus in response to self-antigens, whereas iTregs are peripherally induced from T cells responsible for restraining immune responses to foreign antigens, such as commensal bacteria, food antigens and allergens (16, 17). The mechanism underlying how Tregs are generated remains controversial. Although some studies have suggested that Tregs are anergic to TCR (T cell receptor) stimulation *in vitro*, the process involving the formation and selection of Tregs in the thymus is highly dependent on the TCR rearrangement, as evidenced by the observation that the development of Tregs is abrogated in TCR transgenic mice with RAG-2 deficiency (18). An increasing number of studies have suggested that Tregs are positively selected from autoreactive T cells that express specific TCR with the appropriate affinity for self-peptides (19–21).

Unlike other T helper cells, Tregs lack the capacity to secrete specific cytokines, and it is therefore difficult to distinguish them from other T helper cells. Foxp3 is the most specific Tregs marker and is constitutively expressed in Tregs generated in both the thymus and the periphery irrespective of the mode or state of activation (22, 23). The Foxp3 gene contains 11 exons and maintains a high degree of conservation between human and mouse genes (24). Mice genetically deficient in Foxp3 lose the ability to properly regulate Tregs activity and succumb to a fatal and severe lymphoproliferative autoimmune syndrome at 3–4 weeks of age (25). Similar to mice, humans carrying a FOXP3 mutant gene develop an autoimmune syndrome named IPEX (immune dysregulation, polyendocrinopathy, enteropathy, X-linked syndrome) (26, 27). Beyond its role as an indispensable factor required for the development of Tregs, continuous Foxp3 expression is required for the latter's suppressive function. Research has shown that Tregs isolated from Foxp3 deficient mice lack suppressive function. However, transduction of Foxp3 endows CD4^+^CD25^−^ T cells with the capacity to suppress the proliferation of CD4^+^ T cells (28, 29).

**Figure 1:**
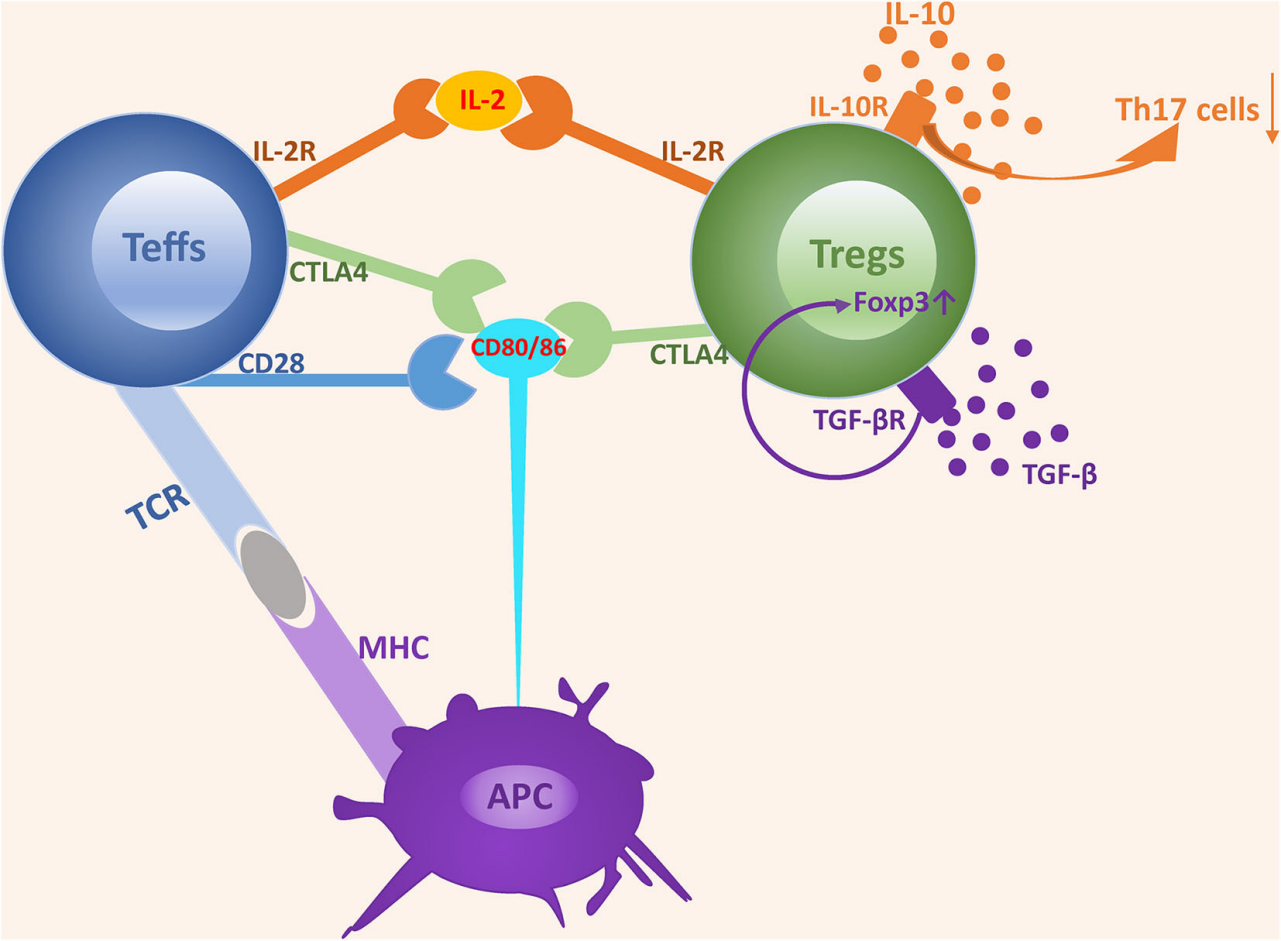
The mechanisms underlying the suppressive function of Tregs. The suppressive function of Tregs is achieved via two mechanisms: cell-contact dependent mechanism and cell-contact independent mechanism. Tregs express a high-affinity IL-2 receptor and can competitively bind to IL-2 with Teffs, which induces IL-2 consumption and suppresses the development and expansion of Teffs. Both CD28 and CTLA4 interact with CD80/CD86 expressed on APCs. However, the affinity of CD28 is lower than that of CTLA4. CD28 plays an important role in enhancing Teffs activation, while CTLA4 acts as an inhibitor by depriving ligands and suppressing CD28 signaling. TGF-β and IL-10 are two classes of nonspecific cytokines secreted by Tregs and can promote Tregs expansion and suppressive activity by binding to their receptors.

The suppressive function of Tregs is achieved via two mechanisms, namely a cell-contact dependent mechanism involving the recognition of co-stimulated molecules that directly suppress the expansion of effector T cells and a cell-contact independent mechanism involving the secretion of soluble cytokines that negatively regulate the immune response (30) (Figure 1).

## Cell-Contact Dependent Mechanism

Cell-contact dependent suppressive activity is mediated via the recognition of co-stimulated molecules. In this process, Tregs function is highly dependent on the normal expression of molecules located on Tregs, and a deficiency of key molecules triggers the defective expansion and suppressive activity of Tregs, leading to a disturbance of immune homeostasis. IL-2 receptor α (IL-2Rα) and CTLA4 are the most important molecules involved in cell-contact dependent mechanism.

Most Tregs abundantly express high-affinity IL-2 receptor α (CD25) and IL-2/IL-2R signaling provides indispensable signaling during the development and maturation of Tregs both in the thymus and in the periphery. Furthermore, the lack of the IL-2R cannot be compensated by other cytokine receptors (31). IL-2, IL-2Rα, and IL-2Rβ deficient mice all die from severe lymphoproliferation and autoimmune disease in early life. In addition, neutralization of circulating IL-2 by anti–IL-2 monoclonal antibodies inhibits Tregs proliferation and triggers a wide range of organ specific autoimmune diseases (32–35). IL-2-IL-2R signaling is essential for the development and maturation of both Tregs and Teff cells, however, low dose IL-2 is remarkably efficacious in promoting the expansion of Tregs rather than Teff cells, which possibly results from the higher affinity of IL-2R in Tregs (36). Based on the comparative activity and different sensitivity for IL-2, the consumption of IL-2 by Tregs has been shown to be a predominant mechanism involved in suppressing the expansion and activity of Teff cells and triggering Teff cell apoptosis due to IL2 deprivation (37, 38).

CTLA4, a key molecule constitutively expressed in Tregs, is crucial for maintaining T cells homeostasis and tolerance induction, and its expression is in part controlled by Foxp3 (39, 40). Mice deficient in CTLA4 become sick by 2 weeks of age and moribund at 3–4 weeks of age, with diffuse and focal lymphocytic infiltration into various organs (41, 42). Furthermore, specific deficiency of CTLA4 in Tregs results in the spontaneous development of systemic lymphoproliferation, multi-organ lymphocyte infiltrations, fatal T cell-mediated autoimmune diseases, and hyperproduction of immunoglobulin E in mice (43). CTLA-4-mediated suppressive regulation of T cell response and upregulation of Tregs activation are predominantly achieved by competition with CD28, a positive costimulatory molecule that shares common ligands (CD80/CD86) with CTLA4 (44, 45). CTLA4 possesses significantly higher affinity in binding CD80/CD86 and CTLA4 rather than CD28 removes costimulatory ligands CD80/CD86 from APCs by a process of trans-endocytosis (46, 47). These properties equip CTLA4 with the capacity to outcompete the ability of CD28 to serve as a negative immune regulator (48, 49).

## Cell-Contact Independent Mechanism

In addition to the cell-contact dependent mechanism, Tregs also exert suppressive activity in a cell-contact independent manner, mainly through the secretion of inhibitory cytokines. Unlike other T cells, Tregs fail to produce exclusive cytokines. However, certain cytokines, such as TGF-β and IL-10, secreted by Tregs are essential for the expansion and suppressive activity of Tregs.

Several lines of evidence suggest that the addition of TGF-β enhances the conversion rate of native T cells into Tregs, and that TGF-β secreted by Tregs plays a partial role in maintaining suppressive properties by binding to the TGF-β receptor (50–53). Administration of neutralizing antibodies specific for TGF-β or specific deficiency of TGF-β expression in Tregs leads to a limitation or even abrogation of Tregs' suppressive activity (54, 55). Strong evidence that the role of TGF-β to maintain Foxp3 expression is supported by the observation that the expression of Foxp3 is dramatically diminished in peripheral Tregs from TGF-β^−/−^ mice and addition of TGF-β results in increased Foxp3 expression (52).

Unlike TGF-β, the function of IL-10 in Tregs seems to be organ-specific. Recent studies have found that IL-10 and IL-35 produced by intratumoral Tregs cooperatively share a common BLIMP1 axis to promote the exhausted intratumoral T cell state and anti-tumor immunity, implying IL-10 and IL-35 contribute to maintaining immune tolerance (56, 57). IL-10 is recognized as a potent suppressor of macrophage and T cell functions. Furthermore, IL-10 deficient mice are growth retarded and suffer from chronic enterocolitis (58). An increasing number of current studies have found that IL-10 is expressed in Tregs and plays an auxiliary role in promoting their expansion and function. IL-10^+^ Tregs are mostly located in intestinal tissues and are essential for limiting immune response-induced inflammation to the diverse intestinal microbiota, which may provide a reasonable explanation as to why IL-10 deficient mice or mice treated with anti-IL-10 receptor blockers succumb to intestinal inflammation (59, 60). Although the Tregs-specific deficiency in IL-10 does not result in severe systemic autoimmunity, it does lead to immunological hyperreactivity at environmental interfaces, resulting in conditions such as spontaneous colitis, lung hyperreactivity, and skin hypersensitivity (61). Thus, while IL-10 production by Treg cells is not necessary for the regulation of systemic autoimmunity, it is essential for hindering excessive immune responses at local environmental interfaces. The suppressive activity of IL-10 is partly mediated via binding to IL-10R to restrain the Th17-induced inflammatory response, which plays a critical role in regulating intestinal homeostasis. This is illustrated by the observation that mice with IL-10R deficient Tregs produce high levels of IL-17 and are prone to developing severe colitis (62, 63).

### Regulation of Fetal-Maternal Tolerance During Healthy Pregnancy

For decades, many studies have shown that successful pregnancy depends on the homeostasis of fetal-maternal tolerance. Furthermore, failure of the maternal immune system to establish fetal-maternal tolerance is the predominant trigger in the development of pregnancy-related complications. Consequently, numerous therapeutic treatments aimed at suppressing the maternal immune system are employed in clinics. However, the effect of these therapies is not always apparent and is often accompanied by various side effects. It is therefore important to identify the cellular and molecular mechanisms responsible for establishing fetal-maternal immune tolerance in healthy and abnormal pregnancies to promote the development of targeted therapeutic interventions. The immune suppressive property of Tregs confers this cell population with a fundamental role in establishing the fetal-maternal immune tolerance necessary for successful pregnancy.

Some studies consider pregnancy to be a process of mutual conversion between pro-inflammatory and anti-inflammatory conditions (64), therefore dividing pregnancy status into three distinct immunological states that correspond to different stages of fetal development: first, a pro-inflammatory stage associated with embryo implantation and placentation (65–67); second, an anti-inflammatory-oriented stage associated with fetal growth (68, 69); and third, a switch from an anti-inflammatory to a pro-inflammatory stage necessary for the initiation of labor (70, 71). Concurrent with the above stages is a dramatic change in the number of Tregs during the course of pregnancy. Following exposure of paternal alloantigens, circulating Tregs increase rapidly during the early pregnancy stage and peak during the second stage at which time trophoblast invasion of the maternal decidua is maximal; then, Tregs gradually decrease when labor begins (64). The change in the number of Tregs and crosstalk with other immune cells play a critical role throughout the entire course of pregnancy.

## Tregs Priming and Implantation

Embryo implantation is the initial stage of pregnancy and involves apposition of the blastocyst and the uterine endometrium followed by attachment and invasion of the blastocyst into the endometrium, and reconstruction of the decidua by the invasive trophectoderm (72). The wide application of assisted reproductive technology, such as *in vitro* fertilization-embryo transplantation (IVF-ET) and intrauterine insemination (IUI), has enabled an analysis of earlier gestational stages from oocyte fertilization to implantation in humans. Adequate endometrial receptivity is considered a pivotal precondition for successful embryo implantation. Endometrial scratching before embryo transfer has been proposed as a clinical treatment to increase uterine receptivity, and some studies have demonstrated that endometrial scratching improves the pregnancy outcome by triggering an inflammatory response and enhancing angiogenesis at the implantation site, providing indirect evidence for the role played by inflammation during implantation (73–77).

Studies based on human and animal experiments have demonstrated that the peri-implantation period is accompanied with the activation and infiltration of various immune cells (78). Uterine-specific natural killer (uNK) cells, macrophages (Mos), and dendritic cells (DCs) are recruited at the implantation site and exert prominent immune-regulatory effects during early pregnancy. uNK cells are the most abundant immune cells located in human decidua during early pregnancy, while Mos and DCs serve as antigen-presenting cells that infiltrate into the decidua. Crosstalk among these cells plays an essential role in regulating trophoblast invasion and in promoting spiral artery remodeling (79–81).

The role played by Tregs during implantation is unclear. However, some studies have reported that a reduced number of Tregs is associated with implantation failure. Mice with a depletion of Tregs exhibit a significant defect in implantation, which is reversed following an adoptive transfer of Tregs (82). A study showed that compared with fertile women, endometrial tissue from women with unexplained infertility displayed a significant reduction Foxp3 mRNA expression, the fate-determining transcription factor especially expressed in Treg cells (83). Other evidence has also revealed a correlation between the level of Tregs in peripheral blood and the implantation rate. Women with implantation failure after IVF or artificial insemination by donor sperm (AID) had a significantly decreased percentage of Tregs compared with women with a successful pregnancy (84, 85). Therefore, the presence of peripheral or local Tregs may create a limited but necessary immunomodulatory function during the course of implantation.

## Enlargement OF Tregs Function and Pregnancy Maintenance

Successful implantation is followed by a phase of fetal growth and development. The establishment of fetal-maternal immune tolerance lays the foundation for this stage, with a shift from a pro-inflammatory immune response to a Th2/Treg-predominant anti-inflammatory immune tolerance (64). The proportion of Tregs begins to rise and peaks at this stage, and a paucity of Tregs could lead to pregnancy-related complications such as spontaneous abortion. Tregs exert a strong immunosuppressive function to maintain an anti-inflammatory environment and protect the fetus from maternal immunological rejection. Tregs can effectively suppress the expansion and activation of effector T cells via a classic cell-contact mechanism or by secreting suppressive cytokines as described previously. One study described a class of functionally distinct Tregs with expression of a co-inhibitory molecule TIGIT, which induces selective suppression of Th1 and Th17 cells but not Th2 cells. However, whether this Tregs subset is expanded and activated during pregnancy is still unknown (86).

The pivotal role played by Tregs in fetal-maternal tolerance raises several questions about the mechanisms responsible for their expansion during pregnancy and underscores the need for studies investigating these mechanisms. Previous studies suggest that the activation and regulation of Tregs is primarily impacted by antigen exposure and the dynamic changes of steroid hormones that occur during pregnancy.

## Antigen-Mediated Tregs Expansion: Paternal Sperm Antigen and Fetal Antigen

Investigators have proposed that exposure to male seminal fluid delivered during mating elicits the expansion of maternal Tregs, as evidenced by the increase in the number of Tregs within the period of time subsequent to mating and before embryo implantation (87, 88). Immune tolerance to the fetus is necessary for successful pregnancy, and transmission of seminal fluid seems to play a priming role prior to implantation by promoting expansion of Tregs, thereby activating specific tolerance to paternal alloantigens. Seminal fluid contains various components, including a cellular fraction that contains sperm, leukocytes and epithelial cells and a non-cellular fraction of compounds such as TGF-β and prostaglandins. The cellular and acellular fractions in semen both contain several antigens, including classical class Ia, non-classical class Ib and minor antigens such as H-Y antigen, which drive an antigen-dependent expansion of Treg cells (89, 90). The non-cellular components are also required to confer tolerance. As mentioned above, TGF-β is a critical cytokine for Tregs proliferation. One study found that intravaginal pre-treatment with TGF-β at mating enhances successful pregnancy *in vivo* in a well-established murine model (91). An *in vitro* experiment also indicated a role for prostaglandins in upregulating Foxp3 expression and enhancing Tregs function (92). Collectively, both sperm and seminal plasma may contribute to driving an expansion of Tregs and providing an immune-privileged environment that is beneficial for subsequent embryo implantation.

Embryo implantation and fetal growth are the most important stages during pregnancy. Some studies have proposed that the implanted blastocyst should be considered a semi-allograft and constant immunosuppression is required for a pregnancy to be successful. Although a seemingly opposite pro-inflammatory process is involved in both implantation and initiation of labor, immunosuppression is an indispensable response to maintain immune homeostasis during the fetal growth stage, and this is highly dependent on the expansion and activation of Tregs triggered by the fetal alloantigens (93). When Tregs are depleted, fetal outcome is normal in syngeneic pregnancies rather than allogeneic pregnancies, suggesting that Tregs suppress maternal immune responses directed against fetal alloantigens rather than male-specific minor histocompatibility antigens (94, 95). When encountered with parental alloantigens presented by a fetus, peripheral Tregs, generated extrathymically and induced by non-self-antigens, serve as the predominant subset suppressing immune response. The development of peripheral Tregs is dependent on the expression of a Foxp3 enhancer CNS1, a deficiency of which leads to an increased resorption of embryos in mice (96).

## Steroid Hormone-mediated Tregs Expansion: Estrogen, Progesterone and Human Chorionic Gonadotropin

**Figure 2:**
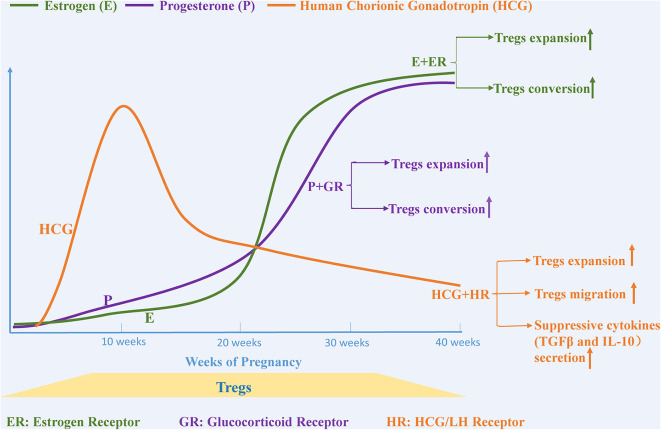
Pregnancy-related hormones affect the expansion and migration of Tregs. The levels of various steroid hormones, such as estrogen, progesterone and human chorionic gonadotropin, change dramatically during pregnancy. Estrogen and progesterone promote Tregs expansion and trigger the conversion of CD4+CD25– T cells to Tregs separately by binding to estrogen and glucocorticoid receptors. The level of human chorionic gonadotropin (HCG), another essential hormone for maintaining a healthy pregnancy, begins to increase after fertilization, peaks at the 11th week, and then gradually decreases until birth. HCG functions as a regulator that not only upregulates the expansion of Tregs but also provokes migration of Tregs from the circulation to the decidua.

Serum levels of the pregnancy-associated hormones such as estrogen, progesterone, and human chorionic gonadotropin (HCG) increase dramatically during pregnancy. These hormones play an essential role in maintaining immune tolerance and in supporting successful pregnancy. Currently, there is increasing evidence that the mechanisms through which hormones contribute to immune homeostasis during pregnancy are in part due to the expansion of Tregs and their suppressive activity (Figure 2).

Estrogen-based therapy has been reported to alleviate symptoms associated with several autoimmune diseases, such as collagen-induced arthritis (97), type1diabetes (98), and autoimmune encephalomyelitis (99). Furthermore, the mechanisms underlying these protective effects seem to be associated with changes in immune cells and cytokines (100–102). The number of Tregs in human peripheral blood change continuously during the menstrual cycle and peak before ovulation, which is concurrent with the change of the concentration of estrogen, suggesting that estrogen may be a powerful factor in promoting Tregs expansion (103). Some studies have demonstrated that the proliferation and suppressive activity of human Tregs observed with estrogen treatment is mediated through estrogen receptor α (104, 105). In both *in vivo* and *in vitro* experiments, estrogen treatment triggers the expansion of Tregs. Furthermore, the addition of estrogen in combination with TCR stimulation enhances Foxp3 mRNA expression in CD4^+^CD25^−^T cells *in vitro*, suggesting that estrogen may potentially induce the conversion of CD4^+^CD25^−^T cells to Tregs (106, 107).

Progesterone, which is mainly produced by the placenta and is markedly elevated during pregnancy, functions as a regulator that maintains homeostasis at the maternal-fetal interface. Similar to estrogen, progesterone is considered to be another important hormone that promotes the expansion of Tregs and their suppressive capacity (107). The proportion of Tregs and the conversion rate of CD4^+^CD25^−^ T cells into Tregs has been shown to increase significantly in the peripheral blood, spleen, and inguinal lymph nodes of ovariectomized mice after progesterone injection (108). Progesterone-mediated immune tolerance is achieved by progesterone binding to the glucocorticoid receptor rather than to the progesterone receptor (109, 110). Progesterone promiscuously binds the glucocorticoid receptor and promotes immune suppression by inducing enrichment of Treg cells and triggering apoptosis of effector T cells, which is based on the preferred sensibility in effector T cells for glucocorticoid receptor-mediated T cells death compared with that in Tregs (110, 111). Progesterone is also present at high levels in human cord blood where it has been reported to have an immune-suppressive function. Progesterone drives a shift of native cord blood T cells into suppressive Tregs, while impeding the conversion from native T cells into Th17 cells, another potential pathway through which progesterone regulates immune tolerance (112).

HCG is another hormone that is increased during pregnancy, and is produced in the blastocyst after fertilization, reaching its maximum level at the 11th week and then gradually decreasing until birth (113). Khil et al. reported that HCG prevents the development of autoimmune-mediated diabetes in NOD mice by downregulating immune effector cells and cytokines and simultaneously upregulating the proportion of Tregs and the levels of TGF-β and IL-10, suggesting that HCG is an effective regulator for immune tolerance (114). Increased HCG during pregnancy provokes many Tregs-related responses including (1) augmenting the number of Tregs, (2) increasing their local and systemic suppressive function, (3) enhancing attraction of circulating Tregs into decidua, and (4) increasing the secretion of suppressive cytokines (115–117). HCG-mediated expansion of Tregs is achieved in part by retaining DCs in an immature state, leading to the generation of Tregs and a loss of the capacity to activate a T cell-mediated immune response (117, 118). *In vitro* migration assays further confirmed the chemoattractant properties of HCG that promote migration of Tregs from the periphery into the uterus, which is potentially mediated via binding to HCG/LH receptors located on Tregs (116, 119).

## Decline of Tregs Activity and Labor

There is a decline in the number of Tregs as pregnancy progresses into the third gestational period. The reduction in the number of Tregs in late gestation may be a contributing factor for the initiation of spontaneous labor. This is supported by the finding that the proportion of Tregs in the decidua following a spontaneous vaginal delivery is significantly lower than that following an elective cesarean section (120). Shah et al. conducted a longitudinal analysis from 20-weeks gestational age to labor and observed a reduction in the number of activated Tregs (defined as Tregs with HLA-DR^+^) and a significant shift toward a Th1/Th17 response with the onset of labor (121). Compared with women undergoing spontaneous term labor, the proportion of activated Tregs is significantly decreased in women in preterm labor (122, 123). The change is similar to the reduction in activated Tregs observed in patients who experience an acute rejection after kidney transplantation, supporting that the reduction in the proportion and activity of Tregs promotes the conversion from an anti-inflammatory to a pro-inflammatory stage and plays a critical role in initiating spontaneous labor. The mechanism underlying the reduction in Tregs during labor remains an enigma. The alteration in hormone levels and in the microbial environment may be stimuli for activating an inflammatory response, however, the specific molecular mechanisms needs to be further investigated.

The level of Tregs progressively decreases after delivery. However, there is a retention of “memory” Tregs with fetal specificity, which retain the ability to generate a more effective and accelerated suppressive response when re-exposed to the same fetal antigens in subsequent pregnancies (124). The primary pregnancy confers Tregs with a protective regulatory memory, which may provide an immunological basis for protection against complications such as pre-eclampsia in a subsequent pregnancy (125, 126).

### Dysfunction of Tregs in Reproductive Diseases

Since it has been determined that Tregs maintain fetal-maternal tolerance during the normal course of embryo implantation and pregnancy, it is of interest to investigate whether systemic and local maldistribution and dysfunction of Tregs play a role in the etiology of infertility and pregnancy-related complications. Increasing evidence suggests that a deficiency in the expansion and function of Tregs as well as an abnormal expression of key molecules are linked to pregnancy-related complications.

## Recurrent Spontaneous Abortion

Recurrent spontaneous abortion (RSA), defined as the loss of three or more consecutive pregnancies, affects ~1% of women attempting to conceive (127, 128). RSA is a complex pregnancy-related complication that is due to multiple factors including chromosomal abnormalities, congenital or acquired anatomical defects in the uterine fundus and cervix, and other endocrine diseases such as PCOS, diabetes, thyroid disorders, and others related to aberrant immune responses (128, 129). Increasing evidences suggests that the proportion of various immune cells and cytokines is altered in patients with RSA, supporting that immune dysfunction may be a contributing factor to its etiology (130, 131). Although there have been detailed guidelines describing clinical interventions for managing women with RSA, treatment based on immune rejection as a potential etiology is controversial, because no definite cellular and molecular mechanism has been discovered to date (17, 129).

The mechanisms through which Tregs contribute to RSA primarily involve an imbalance of the Th1/Th2/Th17/Treg cells paradigm and the abnormal proportion and activity of Tregs. Dysregulation of T lymphocyte homeostasis is also involved in the etiology of RSA. In peripheral blood from patients with RSA, the balance between Th1 and Th2 cells is disrupted in favor of Th1 cells, and the ratio of Th17/Treg cells is skewed toward Th17 cells (132, 133). It is widely accepted that there is a close interaction between the expansion of Tregs and the secretion of IL-17. When IL-17 combines with the IL-17 receptor, Tregs are upregulated. Conversely, Tregs suppress the proliferation of Th17 cells and the secretion of IL-17 via cell-cell contact and via Il-10/TGF-β-mediated effects (134, 135). However, this suppressive function of Tregs is abrogated in patients with RSA (134). Transfusion of Tregs into mice pretreated with IL-17 has been shown to significantly increase the expression of IL-10 and TGF-β, two key cytokines that mediate the suppressive activity of Tregs in decidua and lower the fetal resorption rates in mice (136). Furthermore, insufficient generation of pregnancy-induced Tregs triggers the accumulation of paternal alloantigen specific Th1 cells and directly results in the failure to establish appropriate maternal-fetal immune tolerance (137).

Numerous studies have also confirmed that the reduction in the number of Tregs are involved in the pathogenesis of RSA (Table 1). Sasaki et al. first reported the presence of Tregs in the decidua and demonstrated the proportion of Tregs in decidua from spontaneous abortions was significantly lower than that in decidua from induced abortion (11). Other studies have also demonstrated that the proportion of Tregs and the expression of Foxp3 in both the decidua and peripheral blood from patients with unexplained RSA patients are significantly lower than those from women with normal pregnancies (13, 139). In addition to the reduction in number, Lourdes et al. reported that the suppressive function of Tregs is significantly impaired in RSA as assessed by a co-culture technique with CD4^+^CD25^−^T cells (103). Inadequate number of Tregs and downregulation of Treg cell activity impair the anti-inflammatory environment, weaken the immune tolerance against fetal rejection and thereby increase the risk of RSA.

**Table 1:** The change of the proportion of Tregs in patients with recurrent spontaneous abortion compared with normal pregnant women.

**Proportion of Tregs**		**References**
**Peripheral blood**	**Decidua**	
↓	↓	(11)
↓	↓	(13)
↓	↓	(138)
↓	↓	(139)
↓	Not mentioned	(132)
↓	Not mentioned	(140)
Not mentioned	↓	(141)
↓	Not mentioned	(142)
↓	↓	(143)

*↓: Decreased*.

## Endometriosis

Endometriosis is a benign gynecological disease affecting ~6–10% women of childbearing age, and is characterized by the implantation of endometrial tissues outside the uterus (144). Chronic pelvic pain, dysmenorrhea and infertility are the common symptoms occurring in patients with endometriosis (145, 146). As multiple factors, including genetic and environmental factors, contribute to the development of endometriosis, the pathogenesis of endometriosis remains uncertain. Many theories have been proposed to explain how endometriosis develops, and one of the most widely accepted is the retrograde menstruation theory. This theory hypothesizes that fragments of endometrial tissue reflux to the peritoneum through the fallopian tubes during menstruation (147). However, this theory fails to explain why only a few women develop endometriosis even though retrograde menstruation is a common phenomenon occurring in most women of childbearing age (148). Therefore, other studies have postulated that a disturbed local and systemic immune response may be responsible for the development and progression of endometriosis (149–151).

An aberrant immune environment that includes alternative activation of peritoneal macrophages (152), production of various cytokines (153), and reduction in natural killer cell cytotoxicity (154), all contribute to the survival and invasion of ectopic endometrial tissue. Dysregulation in T lymphocyte homeostasis is associated with the pathogenesis of endometriosis. The Th1/Th2 balance is altered in local and systemic immune conditions, such that there is skewing toward Th2 cells in endometriotic lesions, but skewing toward Th1 cells in peripheral blood (155).

A disturbance in Tregs activity may be a more prominent mechanism involved in the etiology of endometriosis due to their immune-suppressive function, derangement of which could potentially promote the survival of ectopic endometrial lesions. However, evidence regarding the change in the proportion of Tregs in peripheral blood, peritoneal fluid, eutopic endometrium, and ectopic endometrial tissues among patients with endometriosis is inconsistent (Table 2). The discrepancy may result from differences in patient selection, namely the patients with early or advanced endometriosis. Most studies suggest the proportion of Tregs is significantly increased in peritoneal fluid from women with endometriosis compared with control women (157, 158, 161). One study reported that the number of Tregs was increased in the peritoneal fluid and decreased in the peripheral blood, and another study found the number of Tregs was higher in peritoneal fluid than in peripheral blood, both indicating that active translocation of Tregs occurs from circulation to the local peritoneal cavity (158, 162). However, some studies failed to find any difference in the proportion of Tregs in patients with endometriosis when compared with women without endometriosis (159). To bypass the confounding influence of interpatient variability, research has been carried out in an established animal model with endometriosis to identify abnormalities in the proportion of Tregs. In a study of baboons with induced endometriosis, the proportion of Tregs was decreased in peripheral circulation and eutopic endometrium but increased in ectopic tissue, which is consistent with Tregs' local immunosuppressive activity Tregs played (163). Tanaka et al. focused on the variation in resting and activated Tregs and put forth a new concept that the proportion of activated Tregs in the endometrioma rather than in the peritoneal fluid or peripheral blood is decreased, which may be temporal and associated with the angiogenesis and progression of endometriosis (160). However, a study showed the proportion of Tregs in ectopic endometrium was increased in patients with endometriosis compared with eutopic endometrium (156). Further research is required with an expanded sample size and more detailed subgroup analysis to better determine the role Tregs play in the pathogenesis of endometriosis.

**Table 2:** The change of the proportion of Tregs in patients with endometriosis compared with patients without endometriosis.

**Proportion of Tregs in patients with endometriosis**			**References**
Peripheral blood^*^	Peritoneal fluid^*^	**Ectopic peritoneal lesions^#^**	
Not mentioned	Not mentioned	↑	(156)
Not mentioned	↑	Not mentioned	(157)
↓	↑	Not mentioned	(158)
→	→	Not mentioned	(159)
→	Not mentioned	Not mentioned	(155)
→	→	↓	(160)
→	↑	Not mentioned	(161)
→	→ (Early) ↑(Advanced)	Not mentioned	(162)

*↑: Increased, ↓: Decreased, → : Not changed*.

* *The proportion of Tregs in peripheral blood and peritoneal fluid in patients with endometriosis is compared with patients without endometriosis*.

# *The proportion of Tregs in the ectopic peritoneal lesions in patients with endometriosis is compared with eutopic endometrium in patients without endometriosis*.

Change in the proportion of Tregs appears to contribute to the suppressed immune response against ectopic endometrial tissue, permitting implantation of endometrial tissue in the peritoneal cavity. Therefore, understanding the origin of local Tregs production may be provide new insights that will aid in the development of targeted therapies for women with endometriosis. The accumulation of Tregs in the peritoneal cavity may not only be a result of active translocation from the peripheral blood but may also be due to their local induction (153). Higher levels of IL-10 and TGF-β, two key cytokines responsible for regulating the proliferation and activity of Tregs, were found in the peritoneal fluid and serum of patients with endometriosis than in normal controls (164, 165). Compared with serum levels, the level of cytokines in peritoneal fluid was significantly higher (165). Furthermore, IL-10 and TGF-β mRNA expression were significantly higher in ectopic lesions than eutopic endometrium from women with or without endometriosis, particularly in cases of advanced endometriosis (166). These results suggest Tregs and related cytokines maintain the local anti-inflammatory environment and play a crucial role in the development of endometriosis.

## Preeclampsia

Preeclampsia is a common pregnancy-related complication that occurrs in 3–5% of pregnant women and can lead to iatrogenic preterm birth and fetal growth restriction (167). The precise etiology of preeclampsia remains unknown, although insufficient formation of uterine spiral arteries, over-activated inflammation, injured endothelial cells, and genetic factors have all been implicated (168–171). Interestingly, preeclampsia seems to be more common in primiparous than multiparous women, whereas the protective effect is abrogated with the change of partner. A meta-analysis compared the difference in the risk of preeclampsia in women who were impregnated by donor or partner sperm and found the risk was significantly increased in conceptions resulting from donor sperm (172). Furthermore, another study reported that prior and prolonged partner sperm exposure before pregnancy is associated with a significant reduction of the risk of preeclampsia (173). Taken together, these observations suggest that paternal antigens and sperm exposure induce an immune tolerance during the first pregnancy and offer effective protection against the development of preeclampsia with subsequent pregnancies, implying the adaptive immune response with alloantigen specificity and immunological memory is involved in the pathogenesis of preeclampsia (174).

An increasing body of evidence suggests that an inadequate immune tolerance induced by Tregs-associated abnormalities play a pivotal role in the etiology of preeclampsia. Several studies have reported that, compared with normal pregnancy, both the number of Tregs and the ratio of Tregs to Th17 cells in peripheral blood are significantly reduced in preeclampsia (175–177). The increased ratio of Th17/Treg cells has also been confirmed by an analysis of Th17/Treg expression of related transcription factors and the secretion of Th17/Treg-related cytokines. Compared with healthy pregnant women, a reduction in the expression of Treg-specific transcription factor Foxp3 and an elevation in Th17-specific transcription factor RORγt in patients with preeclampsia has been reported (178). Furthermore, analysis of cytokine profiles have revealed a significant decrease in IL-10, and a significant increase in IL-17 levels in patients with preeclampsia (178, 179). Taken together, these studies suggest that a shift occurs from Tregs to Th17 cells in the development of preeclampsia, leading to an abnormal immune state that triggers inflammation and an impairment of immune tolerance. The mechanism underlying the imbalance of Th17/Treg cells remains unclear. Eghbal-Fard et al. suggested the upregulation of miRNA in patients with preeclampsia may affect the differentiation and expansion of Th17/Treg cells by regulating the expression of specific transcription factors (178). In addition to the alteration in the proportion of Tregs, the immunosuppressive activity of Tregs is also altered in patients with preeclampsia. Darmochwal-Kolarz et al. reported the proliferation of effector T lymphocytes in patients with preeclampsia was significantly inhibited by Tregs isolated from healthy pregnant women. However, the suppressive response was lost if replaced with Tregs from patients with preeclampsia (180).

The recruitment of Tregs from peripheral blood into decidua and the local expansion of decidual Tregs are important for maintaining fetal-maternal immune tolerance at the fetal-maternal interface. It has been well-established that the proportion of Tregs in decidua is decreased in preeclampsia (181). Though the reduction of decidual Tregs may be associated with an imbalance in systemic Tregs, local expansion may also play an important role. TCR repertoire analysis of decidual Tregs showed an insufficient clonal expansion of decidual Tregs in preeclampsia compared with healthy pregnancy (182). In normal pregnancy, induced rather than native Tregs are the dominant Tregs subset located in the decidua and are clonally expanded, while the expansive and suppressive capacity of iTregs is significantly impaired in preeclampsia (183). The local induction of Tregs depends on specific APCs within the decidual microenvironment. A significant reduction in the expression of HLA-G and ILT4 on decidual APCs is observed in preeclampsia compared with normal pregnancy, providing a possible clue to the lack of iTregs in preeclampsia (183). An aberrant proportion and type of Tregs in the decidua disturb the immune homeostasis during pregnancy and promote the development of preeclampsia.

### Tregs and Immune Therapy During Pregnancy

Taken together, the above studies suggest that Tregs play a prominent role in regulating fetal-maternal immune tolerance, and a defect in the proportion and activity of Tregs is involved in the development of RSA, endometriosis, and preeclampsia. Thus, approaches designed to boost the proportion of Tregs or strengthen their suppressive function may lead to promising strategies for treating pregnancy-related diseases. Several Tregs-based target therapies are entering into clinical trials, including adoptive Treg cell therapy, Tregs-enhancing drugs, and low dose IL-2 administration (184).

Administration of purified Tregs was firstly applied as Tregs-based target therapy. With the development of immune cell therapy, antigen-specific Tregs therapy was also proposed for treating autoimmune and graft-versus-host diseases. Phase I/II clinical trials aimed to explore the curative effect, and some have reported that Tregs administration alleviates clinical symptoms induced by autoimmunity (184). Some research has attempted to determine whether Tregs administration improves pregnancy outcomes. Yin et al. and Wang et al. examined the effectiveness of adoptive transfer of Tregs in preventing spontaneous abortion in mice models (136, 185). Yin et al. established an abortion-prone pregnancy mice model with DBA/2J-mated pregnant CBA/J mice and performed adoptive transfer of freshly isolated and *in vitro* expanded Tregs from non-pregnant CBA/J mice. Wang et al. induced spontaneous abortions by administration of IL-17 in a CBA/J × BALB/c mouse model of normal pregnancy and performed adoptive transfer of *in vitro* expanded Tregs purified from pregnant CBA/J mice. These two studies demonstrated transfusion with *in vitro* expanded Tregs promotes immune suppressive activity, increases the secretion of suppressive cytokines and significantly reduces the rate of spontaneous abortion.

Although Treg cell therapy has not been widely used in clinical practice, clinical research has initiated several non-specific immunotherapies partially regulating the proportion and activity of Tregs for the treatment of pregnancy-related diseases. Intravenous immunoglobulin G (IVIG) and paternal or third-party lymphocyte immunization therapy have been proposed for the treatment of patients with RSA due to the potential immunomodulatory effects. Although the benefit for these immunotherapies is controversial, a growing body of evidence suggests that they may increase rates of live birth and decrease rates of miscarriage (186–188). A variety of studies and clinical trials have reported both IVIG and lymphocyte immunization therapy correct the Tregs defect and rebalances the Th17/Treg paradigm in peripheral blood. Compared with a control group, the treatment triggers a shift toward Tregs in the Th17/Treg balance by enhancing the expansion of Tregs, promoting the secretion of suppressive cytokines, and inhibiting Th17 cells proliferation (186, 188–192).

Tregs-enhancing drugs are another type of Tregs-based target therapy. Rapamycin (Sirolimus) is an mTOR inhibitor, which acts as an immunosuppressive drug by selectively promoting the expansion of Tregs and inducing differentiation of T helper cells into Tregs. Royster et al. established a murine model with conditional knockdown of Tregs induced by diphtheria toxin. They found the deletion of Tregs decreased litter sizes and triggered embryo implantation failure, effects that were reversed after the treatment with rapamycin (193). A multicenter, double-blind, phase II randomized clinical trial administrated 2 mg/day of sirolimus for 2 days before embryo transfer to patients receiving IVF-ET therapy and who had a history of recurrent implantation failure. The study collected blood samples and assessed the ratio of Th17/Treg cells by flow cytometry 5–10 days prior to the initiation of an IVF cycle. Only patients with a high ratio of Th17/Treg cells were included in this trial. The trial reported that the administration of sirolimus reversed the imbalance in the ratio of Th17/Treg cells and significantly increased the rate of clinical pregnancy and live birth compared with those in the control group (194).

Taken together, some studies have demonstrated the effectiveness of Tregs-based therapy in treating several autoimmune diseases and cases of organ transplantation. However, the methods cannot be directly applied for pregnancy-related diseases because the dynamic change in the immune state during pregnancy and the possibility of fetal drug toxicity must be taken into account. Most of the current treatments for pregnancy-related diseases focus on a reduction in an overactive immune response with the use of non-specific immunosuppressive therapy. This triggers the simultaneous activation of numerous immune cells and makes it difficult to control the dose and to evaluate the curative effect because of individual heterogeneity. Therefore, more studies should be conducted to further explore the effectiveness and safety of Tregs-based target therapies for the treatment of pregnancy-related diseases.

## Conclusion and Future Perspective

Tregs are generally viewed as arising from a specific T cell lineage generated in the thymus or induced in peripheral organs. Being the most predominant immune-suppressive cells, a tremendous amount of research has focused on determining the molecular mechanisms responsible for inducing the expansion of Tregs and their activity in the periphery and in specific organs. This effort will provide new insights that will guide the improvement of Tregs-based targeted immune therapy. In recent years, increasing data has shown that the expansion of Tregs is triggered after exposure to the fetal alloantigens and changes dynamically over the course of pregnancy. Hormones such as estradiol and progesterone as well as HCG are significantly increased during pregnancy, and regulate the number and function of Tregs to sustain a proper pregnancy-related immune tolerance. Furthermore, various reproductive diseases such as recurrent miscarriage, endometriosis and preeclampsia result in part from the deficiency in the number and activity of Tregs. Therefore, modulating the immune response by boosting the number of Tregs and enhancing their activity may be a potential therapeutic strategy for managing these pregnancy-related complications.

## Author Contributions

All authors listed have made a substantial, direct and intellectual contribution to the work, and approved it for publication.

## Conflict of Interest

The authors declare that the research was conducted in the absence of any commercial or financial relationships that could be construed as a potential conflict of interest.
